# *BRAF* mutations in non-Hodgkin's lymphoma

**DOI:** 10.1038/sj.bjc.6601371

**Published:** 2003-11-11

**Authors:** J W Lee, N J Yoo, Y H Soung, H S Kim, W S Park, S Y Kim, J H Lee, J Y Park, Y G Cho, C J Kim, Y H Ko, S H Kim, S W Nam, J Y Lee, S H Lee

**Affiliations:** 1 Department of Pathology, College of Medicine, The Catholic University of Korea, 505 Banpo-dong, Socho-gu, Seoul 137-701, Korea; 2 Department of Diagnostic Pathology, Samsung Medical Center, Sungkyunkwan University, Seoul 135-710, Korea

**Keywords:** mutation, BRAF, RAS, non-Hodgkin's lymphoma, oncogene

## Abstract

Ras proteins control signalling pathways that are key regulators of several aspects of normal cell growth and malignant transformation. *BRAF*, which encodes an RAF family member in the downstream pathway of RAS, is somatically mutated in a number of human cancers. The activating mutation of *BRAF* is known to play a role in tumour development. As there have been no data on the *BRAF* mutation in non-Hodgkin's lymphoma (NHL), we analysed the genomic DNAs from 164 NHLs by polymerase chain reaction (PCR)-based single-strand conformation polymorphism (SSCP) for the detection of somatic mutations of *BRAF* (exons 11 and 15). Overall, we detected *BRAF* mutations in four NHLs (2.4%). Whereas most *BRAF* mutations in human cancers involved V599 of BRAF, all of the four *BRAF* mutations in the NHLs involved other amino acids (one G468A, two G468R and one D593G). To our knowledge, this is the first report on *BRAF* mutation in NHL, and the data indicate that BRAF is occasionally mutated in NHL, and suggest that *BRAF* mutation may contribute to the tumour development in some NHLs.

The activated RAS proteins excite the mitogen-activated protein (MAP) kinase pathway (RAS–RAF–MEK–ERK–MAP kinase pathway) by recruiting the cytosolic protein RAF (Downward, 2003). *RAF* gene family consists of three members (*ARAF1*, *BRAF* and *RAF1*), each encoding serine/threonine kinases that are regulated by binding to RAS. RAS–RAF–MEK–ERK–MAP kinase pathway plays a critical role in cell proliferation, and is frequently activated in cancer cells. For example, approximately 10–20% of all human tumours contain mutated versions of RAS proteins (Bos, 1989). Recently, Davies *et al* (2002) identified somatic mutations of *BRAF* in 66% of malignant melanomas and at a lower frequency in a wide range of human cancers. So far, all *BRAF* mutations have been reported within two kinase domains (G-loop and activation segment domains), and the most common mutation is a single substitution, V599E (Brose *et al*, 2002; Davies *et al*, 2002; Naoki *et al*, 2002; Rajagopalan *et al*, 2002; Yuen *et al*, 2002; Pollock *et al*, 2003; Satyamoorthy *et al*, 2003). Mutated BRAF proteins have elevated kinase activity and transforming activity in NIH3T3 cells (Davies *et al*, 2002). Furthermore, RAS function is not required for the growth of cancer cell lines with the V599E mutation (Davies *et al*, 2002). Several studies have reported a low incidence of *Ras* gene mutation in non-Hodgkin's lymphoma (NHL) (Ahuja *et al*, 1990). Although screening of *BRAF* mutation in human tumours has widely been performed, to date the data on *BRAF* mutation in NHL tissues is lacking. In the present study, we investigated the occurrence of *BRAF* gene mutations in NHLs.

## MATERIALS AND METHODS

Paraffin-embedded tissues of human NHL were obtained from 164 patients. These samples were stained with haematoxylin–eosin, examined by immunohistology, and then classified according to the Revised European-American Lymphoma (REAL) classification (Harris *et al*, 1994). The NHLs analysed consisted of seven B-cell small lymphocytic lymphomas, three mantle cell lymphomas, four follicular lymphomas, 49 mucosa-associated lymphoid tissue (MALT)-type lymphomas, 67 diffuse large B-cell lymphomas, four precursor T-lymphoblastic lymphomas, one T-cell chronic lymphocytic leukaemia, 14 peripheral T-cell lymphomas, unclassified, 14 angiocentric lymphomas, and one intestinal T-cell lymphoma. Ethical committee approval for the study was obtained. Through the microdissection technique, we selectively procured tumour cells and corresponding normal cells from histological sections of the 164 NHLs. Briefly, malignant cells were selectively procured from haematoxylin and eosin-stained sections using a 30G1/2 hypodermic needle (Becton Dickinson, Franklin Lakes, NJ, USA) affixed to a micromanipulator, as described previously (Lee *et al*, 1998). We also microdissected normal cells and used them for corresponding normal DNA. This microdissection technique used in this study has been proved to be precise and effective for procurement of tumour cells without normal cell contamination (Lee *et al*, 1998). DNA extraction was performed by a modified single-step DNA extraction method, as described previously (Lee *et al*, 1998).

Genomic DNA each from normal cells or tumour cells was amplified with two primer pairs covering exons 11 and 15 of *BRAF* gene, because all of the *BRAF* mutations have been so far detected in exons 11 and 15 that encode the kinase domains in G-loop and the activation segment of BRAF, respectively. Radioisotope was incorporated into the PCR products for detection by autoradiogram. The PCR reaction mixture was denatured for 1 min at 94°C and incubated for 30 cycles. Other procedures of polymerase chain reaction (PCR) and single-strand conformation polymorphism (SSCP) analysis were performed as described previously (Shin *et al*, 1999). After SSCP, DNAs showing mobility shifts were cutout from the dried gel, and reamplified for 30 cycles using the same primer sets. Sequencing of the PCR products was carried out using the cyclic sequencing kit (Perkin-Elmer, Foster City, CA, USA) according to the manufacturer's recommendation.

## RESULTS

**Figure 1:**
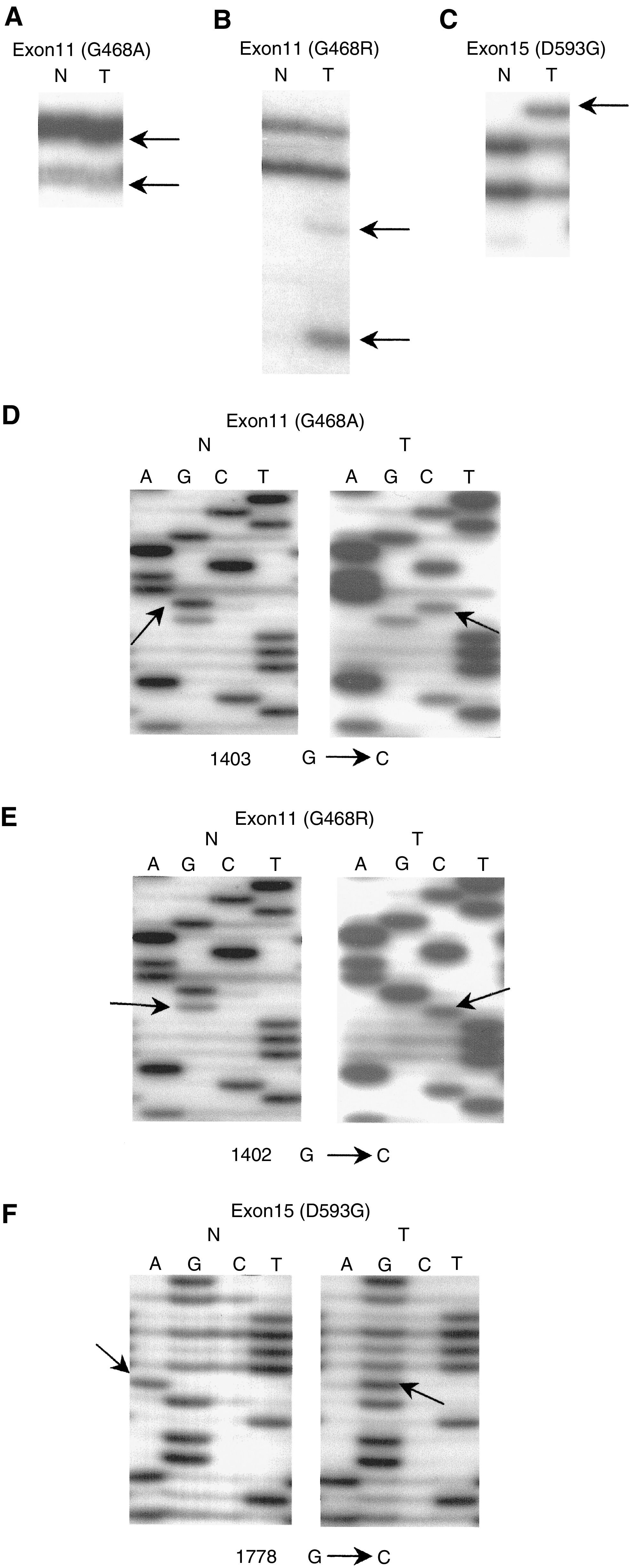
*BRAF* gene mutations in NHLs. SSCP (**A**–**C**) and DNA sequencing analyses (**D**–**F**) of DNA from tumours (lane T) and normal tissues (lane N). Exon 11 (**A**, **B**) and exon 15 (**C**) of *BRAF* were amplified. SSCPs of DNA from the tumours show wild-type bands and additional aberrant bands (arrows) as compared to SSCP from normal cells from the same patients. (**D**) Sequencing analysis from the aberrant band in (**A**). There is a G to C transversion at nucleotide 1403 of *BRAF* (arrow) in tumour tissue as compared to normal tissue. (**E**) Sequencing analysis from the aberrant band in (**B**). There is a G to C transversion at nucleotide 1402 of *BRAF* (arrow) in tumour tissue as compared to normal tissue. (**F**) Sequencing analysis from the aberrant band in (**C**). There is an A to G transition at nucleotide 1778 of *BRAF* (arrow) in tumour tissue as compared to normal tissue. Numbering of cDNA of *BRAF* was made in respect to the ATG start codon (GenBank).

SSCP analysis of *BRAF* identified four aberrant bands (Figure 1

). Enrichment and DNA sequence analysis of these aberrantly migrating bands led to the identification of four *BRAF* mutations (2.4%) (Figure 1). All of the four *BRAF* mutations were observed in diffuse large B-cell lymphomas (6.0% of the 67 cases). Although *BRAF* mutations were detected only in diffuse large B-cell lymphomas, this relationship was not statistically significant (*P*>0.05). Three of the four *BRAF* mutations involved codon 468 (two G468R and one G468A) in the G-loop domain, and the remaining one was found at codon 593 (D593G) in the activation segment domain (Table 1

**Table 1:** Summary of *BRAF* mutations identified in the NHLs

**BRAF mutations**			
**Nucleotide**	**Amino acids**	**Anatomical site**	**Histologic type**
G1403C	G468A	Cervical lymph node	Diffuse large B-cell lymphoma
G1403C	G468A	Cervical lymph node	Diffuse large B-cell lymphoma
G1402C	G468R	Tongue mucosa	Diffuse large B-cell lymphoma
A1778G	D593G	Ileum	Diffuse large B-cell lymphoma

, Figure 1). None of the corresponding normal samples showed evidence of mutations by SSCP (Figure 1), indicating the mutations detected in the specimens had risen somatically. We repeated the experiments two times, including tissue microdissection, PCR, SSCP and sequencing analysis to ensure the specificity of the results, and found that the data were consistent (data not shown).

## DISCUSSION

Whereas the malignant melanoma is the most common tumour with *BRAF* mutations (roughly 60%), this tumour is known to possess a much lesser frequency of *RAS* mutations. Such differential occurrences of *BRAF* and *RAS* mutation in some human cancers led us to analyse *BRAF* mutation in NHL in which *RAS* mutation is known to be an uncommon event. We found that *BRAF* gene is somatically mutated in NHLs, indicating that RAS–RAF kinase pathway in some NHLs may be regulated by somatic mutations of *BRAF*. Despite the low frequency of *BRAF* mutation in NHL compared with that of malignant melanoma, our data suggest that alteration of RAS–RAF kinase pathway by *BRAF* mutation may play an important role in NHL carcinogenesis.

In the present study, none of the *BRAF* mutations involved the amino acid V599. The data are quite contrast to those of malignant melanomas, where approximately 90% of *BRAF* mutations involved V599, raising the possibility that the contribution of *BRAF* mutations in the development of NHL might be different from that of malignant melanoma. Additionally, three (two G468A and one G468R) of the four *BRAF* mutations in this study involved the same amino acid (G468) that is located in the GXGXXG motif within the G-loop of the kinase domain. The G468A mutation was proven to be an activating mutation by the kinase assay and the transformation assay (Davies *et al*, 2002). For G468R, a novel *BRAF* mutation, its functional implication is not known at this stage. In one NHL, we also found D593G *BRAF* mutation that has also been detected in colon tumours previously.

The most impressive examples of recent cancer therapies used protein kinase inhibitors such as Imanitib (Gleevec) (Downward, 2003). Since RAS–RAF–MEK–ERK–MAP kinase pathway is activated by protein kinase, therapies that target this signalling pathway would therefore be very valuable in treating tumours that have activating mutations of *BRAF*. In this respect, the present study may provide the possibility of therapy targeting mutated BRAF in NHL.
